# An evaluation of new and established methods to determine T‐DNA copy number and homozygosity in transgenic plants.

**DOI:** 10.1111/pce.12693

**Published:** 2016-01-21

**Authors:** Katarzyna Głowacka, Johannes Kromdijk, Lauriebeth Leonelli, Krishna K. Niyogi, Tom E. Clemente, Stephen P. Long

**Affiliations:** ^1^ Carl R. Woese Institute for Genomic BiologyUniversity of Illinois1206 W Gregory DriveUrbanaIL61801USA; ^2^ Institute of Plant GeneticsPolish Academy of Sciencesul. Strzeszyńska 3460‐479PoznańPoland; ^3^ Howard Hughes Medical Institute, Department of Plant and Microbial Biology, 111 Koshland HallUniversity of California BerkeleyBerkeleyCA94720‐3102USA; ^4^ Molecular Biophysics and Integrated Bioimaging DivisionLawrence Berkeley National LaboratoryBerkeleyCA94720USA; ^5^ Center for Plant Science InnovationE324 Beadle Center, 1901 Vine StreetLincolnNE68588USA

**Keywords:** ddPCR, digital droplet PCR, qPCR, segregation analysis, selectable marker, Southern blot, TAIL‐PCR, transformation

## Abstract

Stable transformation of plants is a powerful tool for hypothesis testing. A rapid and reliable evaluation method of the transgenic allele for copy number and homozygosity is vital in analysing these transformations. Here the suitability of Southern blot analysis, thermal asymmetric interlaced (TAIL‐)PCR, quantitative (q)PCR and digital droplet (dd)PCR to estimate T‐DNA copy number, locus complexity and homozygosity were compared in transgenic tobacco. Southern blot analysis and ddPCR on three generations of transgenic offspring with contrasting zygosity and copy number were entirely consistent, whereas TAIL‐PCR often underestimated copy number. qPCR deviated considerably from the Southern blot results and had lower precision and higher variability than ddPCR. Comparison of segregation analyses and ddPCR of T_1_ progeny from 26 T_0_ plants showed that at least 19% of the lines carried multiple T‐DNA insertions per locus, which can lead to unstable transgene expression. Segregation analyses failed to detect these multiple copies, presumably because of their close linkage. This shows the importance of routine T‐DNA copy number estimation. Based on our results, ddPCR is the most suitable method, because it is as reliable as Southern blot analysis yet much faster. A protocol for this application of ddPCR to large plant genomes is provided.

## Abstract 2

Genetic transformation is being used increasingly in the public domain to test a range of hypotheses concerning gene action, not only in Arabidopsis, but now in a broad range of plants. A major challenge though, particularly with species with relatively long life cycles, is in identifying individuals that are homozygous for the insert or DNA modification at T2, which is necessary to provide homozygous lines. Southern blotting has been the traditional approach, but it is slow and requires considerable skill. Various PCR methods have been used to accelerate testing, but have not been as reliable. However, we show here that the recently developed digital droplet PCR is as effective as Southern blotting, yet faster and capable of high‐throughput. A protocol for application of ddPCR is provided together with evidence of its efficacy.

## Introduction

The use of stable transformation of plants to test a range of hypotheses has been increasing rapidly and has been suggested a key element in addressing future security of food supply as well as in adapting to global change (Khush 2005; Hibberd *et al.* 2008; Long *et al.* 2015). In parallel, there is considerable interest in introducing new pathways and modifying existing pathways in plants to produce new or improved bioproducts (Clemente & Cahoon 2009; Rogers & Oldroyd 2014). Rapid and reliable evaluation of transgenic allele(s) for copy number and homozygosity is a vital step in utilizing these transformation events, so that homozygous lines with stable transgene expression are obtained for accurate testing. Knowledge of the transgenic locus structure and zygosity status is particularly important in plants, including all crops, which have a life cycle of months and sometimes years. Here, decreasing the number of generations between the initial transformation and identification of homozygous lines with stable expression has greatest value.

Southern blot analysis (Southern, 1975), quantitative polymerase chain reaction (qPCR; Higuchi *et al.* 1992), thermal asymmetric interlaced‐PCR (TAIL‐PCR; Liu *et al.* 1995) and most recently digital droplet PCR (ddPCR; Hindson *et al.* 2011) have all been used to provide information about the integration status of a transgenic allele(s) in genomes (Tingay *et al.* 1997; Fu *et al.* 1999; Campbell *et al.* 2000; Ingham *et al.* 2001; German *et al.* 2003; Pillai *et al.* 2008; Mieog *et al.* 2013; Yi *et al.* 2013; Larkan *et al.* 2016; Wang *et al.* 2015). However, these methods use contrasting principles for characterization of a transgenic allele and differ vastly in precision, reproducibility and potential to scale up. Southern blot analysis has been regarded as the most unambiguous method for molecular characterization of transgenic alleles for both estimation of copy number and loci complexity in transgenic plants. The disadvantage is that the process is more labour intensive, and less well suited for automation, relative to PCR‐based platforms. Here the precision and speed of different PCR methods are compared to Southern blot analysis.

Typically, a large number of independent transformations are generated to compensate for insertions in undesirable genomic loci; for example insertion into a native open reading frame, or in a region not conducive to stable expression levels. The resulting collection of primary transformants can then be screened to prioritize lead events that possess targeted transgene expression levels, coupled with simple integration and low copy transgenic allele(s). Importantly, an ideal genotyping platform should possess the ability to facilitate identification of homozygous lineages in early generations of selection.

In Southern blot analysis (Southern, 1975) genomic DNA is digested, separated on a gel, blotted onto a membrane and then detected with a radioactive, fluorescent or chemi‐luminescent labelled probe sequence to visualize the transgenic allele(s) complexity of integration. The intensity of the hybridization signal in reconstruction lanes, whereby known quantities of the target sequences are spiked into native DNA, is correlative with zygosity at each allele (Dai *et al.* 2001). In qPCR (Higuchi *et al.* 1992), template concentration is estimated based on the fluorescence trace from a dye or probe in the sample which is directly proportional to the template amplification. Copy number, as well as zygosity, of the transgenic allele(s) can be derived from qPCR analysis, but logarithmic PCR template quantification may limit ability to identify small copy number differences (Bubner & Baldwin 2004). Additionally, direct coupling between the PCR amplification and quantification makes qPCR very sensitive to PCR efficiency (Bustin *et al.* 2009). TAIL‐PCR (Liu *et al.* 1995) has also been used to establish the number of insertions in transgenic events by means of amplifying the flanking sequence of the transgenic allele(s), which can be exploited to map the allele if a genome sequence is available (e.g. Hanhineva & Kärenlampi 2007). Recently, a ddPCR method has been developed (Hindson *et al.* 2011) which can also be used to detect absolute DNA copy concentration at high accuracy. The detection principle is based on a fluorescent dye or probe, similar to qPCR. However in ddPCR, the PCR reaction is rendered digital by randomization of the sample DNA over a set of subsamples, in which the DNA dilution is chosen to obtain both positive and negative subsamples for template presence. A Poisson probability distribution can then be used to derive the template concentration (Hindson *et al.* 2011). Decoupling of amplification and quantification should make ddPCR results relatively insensitive to PCR efficiency and the linearity of the quantification scale should allow detection of small copy differences (Hindson *et al.* 2013, Bharuthram *et al.* 2014). While segregation analysis of transgene presence in subsequent generations can provide information about the mode of inheritance, and a means to identify homozygous lines for down‐stream phenotyping, this approach requires several generations of inbred plants to be analysed and associated costs for time, space and labour can be substantial. Additionally, although *Agrobacterium tumefaciens*‐mediated transformation typically results in a lower T‐DNA copy number than direct DNA transfer methods such as electroporation or particle bombardment, multiple T‐DNAs integrated at the same locus can still be found (Jorgensen *et al.* 1987; Kohli *et al.* 1999; Gelvin 2003). Such multiple inserts at a single locus would not be detected by segregation analysis. Multiple T‐DNA copies can increase the likelihood of silencing effects, in particular when tandem T‐DNA insertions are arranged in inverted repeats (Jorgensen *et al.* 1987; Stam *et al.* 1997). This makes detecting these multiple inserts of particular importance in selecting for stable expression.

The aim of this study was to assess both the importance of estimating copy number and to assess which method is most suited to estimating copy numbers and establishing homozygosity in transgenic plants. T‐DNA copy number in the same transgenic plants was evaluated with Southern blot, qPCR, TAIL‐PCR and ddPCR analyses. Subsequently we assessed reproducibility of parallel qPCR and ddPCR analyses, using both methods with 12 different primer combinations on the same DNA sample. After comparing data output, duration of protocol, reproducibility and precision, the ddPCR method appeared most suitable to routinely assess T‐DNA copy numbers and homozygosity. An example protocol optimized for use in higher plants with large genomes is provided in the Supporting Information.

## Material and Methods

### Plant material and transformation

*Nicotiana tabacum* cv. ‘Petite Havana’ was transformed using the *Agrobacterium*‐mediated leaf disc protocol according to Clemente (2006), a protocol based on the seminal communication by Horsch *et al.* (1985), using two different plasmids. The first plasmid (PsbS) contained the coding sequence of the photosystem II subunit S gene from *Nicotiana benthamiana* (*Nb*PsbS). Overexpression of PsbS results in a proportional increase in non‐photochemical quenching (NPQ) of chlorophyll fluorescence in transformed plants (Li *et al.* 2002). Therefore chlorophyll fluorescence analysis could be used to identify transformed individuals in segregation analysis. The second plasmid (VPZ) contained coding sequences of three genes from *Arabidopsis* *thaliana*: violaxanthin de‐epoxidase (*At*VDE), *At*PsbS and zeaxanthin epoxidase (*At*ZEP). T‐DNA maps are provided in Supporting Information (Fig. S1A–B). The presence of three genes in transformants with construct 2 allowed us to verify reproducibility of the qPCR and ddPCR method with multiple primer sets to derive the copy number of the transgenic cassette. Additionally, both constructs contained the *bar* gene encoding resistance for bialaphos (Thompson *et al.* 1987). Multiple confirmed independent T_0_ transformants were generated for each construct (11 and 15 for PsbS and VPZ, respectively).

### DNA extraction for PCR methods

Young leaf tissue was collected from greenhouse‐grown plants to determine T‐DNA insert copy number, snap frozen in liquid nitrogen and stored in −20 °C. DNA was extracted by the CTAB method modified from Kabelka *et al.* (2002).

### Southern blot

Southern hybridization was carried out on a subset of eight plants derived from one VPZ transformation event (VPZ‐23) representing T_0_, T_1_ and T_2_ generations. These were used to compare Southern blot analysis with the three PCR based methods. Genomic DNA was extracted using a modified version of the protocol by Dellaporta *et al.* (1983). Fifteen micrograms of genomic DNA was digested overnight with *Bam*HI (R3136, New England Biolabs, Ipswich, MA, USA) and separated on a 0.8% agarose gel at 25 V overnight and alkali blotted onto a Zeta‐probe GT genomic tested blotting membrane according to the manufacturer's specifications (BioRad, Hercules, CA, USA). Probe DNA was obtained by double digestion of the VPZ plasmid using *Xho*I (R0146, New England Biolabs, Ipswich, MA, USA) and *Xba*I (R0145, New England Biolabs, Ipswich, MA, USA), yielding a 594 bp fragment, which was radiolabelled with α‐^32^P‐labelled dCTP by means of the Prime‐It II random primer labeling kit according to the manufacturer's protocol (Stratagene, La Jolla, CA, USA). Pre‐hybridization and hybridization were carried out at 65 °C in the presence of high salt buffer following the manufacturer specifications. Membranes were analysed by autoradiography (X‐Omat AR5 film, Eastman Kodak, Rochester, NY, USA).

### Thermal asymmetric interlaced PCR (TAIL‐PCR)

DNA for thermal asymmetric interlaced (TAIL‐) PCR was extracted as described above. TAIL‐PCR conditions were the same as described in Liu *et al.* (1995), except that primary and secondary reaction volumes were 25 *μ*L instead of 20 *μ*L and tertiary reaction volumes 50 *μ*L instead of 100 *μ*L. Additionally, in all three reactions the Phusion high fidelity PCR master‐mix with high fidelity buffer (M0531S, New England Biolabs, Ipswich, MA, USA) was used together with appropriate primer set with 2 *μ*M of arbitrary degenerate primer (AD1, AD2 or AD3) and 200 nmole of T‐DNA specific primer (TR1, TR2 or TR3; for primer sequences see Table S1). The amplicons of all three reactions were separated on 1% agarose gels (General purpose agarose GP2, BE‐A125, MidSci, Valley Park, MO, USA) with Tris‐Borate‐EDTA buffer and visualized with ethidium bromide.

### Quantitative PCR (qPCR)

DNA obtained as described above was digested overnight with *Hind*III (R3104, New England Biolabs, Ipswich, MA, USA), followed by purification with cleaning and concentrating columns (D4014, Zymo Research, Irvine, CA, USA). Reactions were prepared using 9 *μ*L of digested genomic DNA (20–25 ng *μ*L^−1^), 200 nmole of forward and reverse primersets AtPsbS_3, AtPsbS_4, AtVDE_1, AtVDE_4, AtZEP_1 or AtZEP_4 for T‐DNA amplicons and NtActin_1 and NtTubulin_1 for reference genes (for primer sequences, see Supporting Information Table S1) and 10 *μ*L of SsoAdvanced Universal SYBER Green Supermix (172‐5270; BioRad, Hercules, CA, USA). The reaction efficiency for each primer set was estimated on a series of DNA dilutions. All reactions were run on CFX connect Real‐Time PCR Detection System (1855201, BioRad, Singapore) using the following program: 3 min 95 °C, 40 times (10 s 95 °C; 30 s 60 °C), followed by melting curve generated from 65 °C to 90 °C. Four technical replicates were used for both T‐DNA and the reference sequence. Raw data was processed using BioRad CFX Manager 3.1 and T‐DNA copy number estimates made using the ΔΔCt method (Livak & Schmittgen, 2001) and corresponding standard errors were computed as described in Hoebeeck *et al.* (2007).

### Digital droplet PCR (ddPCR)

Digested DNA was obtained as described for qPCR, excepting that in addition to HindIII. SacI (R3156, New England Biolabs, Ipswich, MA, USA) was also used for the digestion of DNA derived from PsbS transformants. The full step‐by‐step protocol for ddPCR is described in the Supporting Information (Appendix 1). Briefly, the ddPCR reactions were prepared using 11.5 *μ*L of digested genomic DNA (20–25 ng *μ*L^−1^), 100–150 nmole of forward and reverse primers (for primer sequences, see Supporting Information Table S1), 12.5 *μ*L of a commercial reaction mix including an intercalating fluorescent dye, polymerase, Mg^2+^ and dNTPs (2x QX200 ddPCR EvaGreen Supermix, 186‐4034, BioRad, Hercules, CA, USA) and MilliQ autoclaved water (dMQ H_2_O) to a total reaction volume of 25 *μ*L. Droplets were generated using droplet generator cartridges, gaskets and cartridge holder (186‐4007 and DG8, 186‐3051, Bio‐Rad, Hercules, CA, USA) in a droplet generator (QX200, 186‐4002, Bio‐Rad, Hercules, CA, USA). According to the manufacturer's instructions, 20 *μ*L of ddPCR reaction mix and 70 *μ*L of droplet generation oil (EvaGreen 186‐4005, Bio‐Rad, Hercules, CA, USA) were loaded into cartridges. The resulting 40 *μ*L sample of generated droplets was dispensed to one well of a 96‐well PCR semi‐skirted plate (951020362, Eppendorf, Enfield, CT, USA) and sealed with foil (Pierceable foil heat seal, 181‐4040 and Px1 PCR plate sealer, 181‐4000, Bio‐Rad, Hercules, CA, USA ). The droplet mix was cycled through a PCR program using a deep‐well thermal cycler (C1000 Touch, 185‐1196, Bio‐Rad, Hercules, CA, USA), followed immediately by analysis in a droplet reader (QX200, 186‐4003, Bio‐Rad, Hercules, CA, USA).

Fluorescence reads per individual droplet from each well were analysed with the manufacturer‐provided software (Quanta Soft version 1.7, 1864011, Bio‐Rad, Hercules, CA, USA). The droplet population in each well was divided into template negative or positive. The distribution of positive and negative droplets is a function of the starting PCR template concentration according to the Poisson probability distribution, which allows the absolute concentration of PCR‐template to be derived from the number of negative droplets according to Eqn 1:

(1) $$PCR\;\text{Concentration}-\text{template}\;=\frac{-ln\left({}^{\text{Nneg}}/N\right)}{V}$$

where *N_neg_* is the number of negative droplets; *N* is the total number of droplets and *V* is volume of a single droplet which is equal to 0.85 nL. For the calculation of copy number the ratio between the concentration of the T‐DNA and a reference sequence of known copy number was used. Two primer sets were used to amplify reference sequences in either α‐tubulin (two copies) or actin (four copies). Primer sequences were designed to amplify regions in contig c61851 and c50972, respectively from *N. tabacum* (Methylation Filtered Genome TGI: v.1 Contigs; accessed August 4, 2014). The performance of each primer set was empirically verified prior to use. T‐DNA copy number and corresponding standard errors were calculated based on the ratio of T‐DNA versus reference sequence according to Hedges *et al.* (1999). Two technical replicates were used for both T‐DNA and reference gene ddPCR reactions. In all comparative analyses, the same primers were used for qPCR and ddPCR.

### Segregation analysis according to glufosinate ammonium resistance

T_1_ progeny of 11 PsbS lines and 15 VPZ lines were used in segregation studies of resistance to glufosinate ammonium. Seeds collected from T_0_ plants were germinated in round 20‐cm‐diameter pots (ITML Inc, Brantford, ON, Canada) containing a peat‐, bark‐ and perlite‐based growing medium (Metro‐Mix 900, Sun Gro Horticulture, Agawam, MA, USA). Five days after germination, 24 randomly chosen seedlings of each line were transferred to twelve 4 cm × 3 cm × 5.7 cm cells of planting trays (715364C, T.O. Plastics, MN, USA) placed on flats (710251C, T.O. Plastics, MN, USA) filled with a growing medium with a reduced perlite content (Metro‐Mix 510, Sun Gro Horticulture, Agawam, MA, USA). Seedlings were grown in a controlled environment walk‐in growing chamber with 12 h day (23 °C)/12 h night (18 °C) cycle under 150 *μ*mol quanta m^‐2^ s^‐1^. Ten days after transplanting, seedlings were sprayed with 1.5 g L^‐1^ glufosinate ammonium solution [0.8% (*v/v*) Ignite herbicide, cas77182‐82‐7, Bayer CropScience, Research Triangle Park, NC, USA]. Presence of necrotic and chlorotic leaf tissue was assessed 10 days after exposure and used to score plants as either susceptible or resistant. Additionally, 72 plants of each of five T_1_ lines were grown in the field, and mature leaves of each plant were painted with the same solution and then assessed for damage 10 days later. In both cases, the observed susceptible plant counts were compared against the expected counts based on 1:3 segregation using a Chi^2^ test (*α* = 0.1).

### Segregation analysis using NPQ level in PsbS‐transformed plants

The abundance of PsbS protein is tightly linked to the maximum level of rapidly reversible NPQ of Photosystem II (NPQ; Li *et al.* 2002). This property allows use of NPQ level as an independent marker for progeny segregation. Ten‐day‐old seedlings of T_1_ progeny of 11 PsbS lines were germinated and grown, as described in the preceding section, and used in modulated chlorophyll fluorescence imaging to determine NPQ (CF Imager, Technologica, Colchester, UK). Seedlings were dark adapted for 20 min and then the dark‐adapted maximal fluorescence (Fm) was imaged with a 800 ms saturating flash (6000 *μ*mol quanta m^‐2^ s^‐1^) from blue LEDs (*λ*_max_ = 470 nm). Immediately following the dark‐adapted reading, blue LEDs were powered to provide 1000 *μ*mol quanta m^‐2^ s^‐1^ on the seedlings. After 10 min a second saturating flash was applied to determine the maximum fluorescence under illuminated conditions (Fm′). Average NPQ per seedling was then calculated from these measurements according to Eqn 2, assuming the Stern–Volmer quenching model (Maxwell and Johnson 2000):

(2) NPQ=Fm/Fm’−1.

To separate transgenic T_1_ progeny into those carrying the PsbS construct or segregated back to wild‐type (WT), the mean and standard deviation of WT seedling NPQ values were used to identify a threshold level of NPQ, above which seedlings were classified as transgenic (*α* ≤ 0.05). The resulting transgenic and WT counts for each T_1_ progeny were subsequently used to estimate the number of independently inherited loci, by comparing against the expected counts based on 1:3 segregation using a Chi^2^ test (*α* = 0.1).

## Results

### Comparison between southern blot analysis, TAIL‐PCR, qPCR and ddPCR

**Figure 1:**
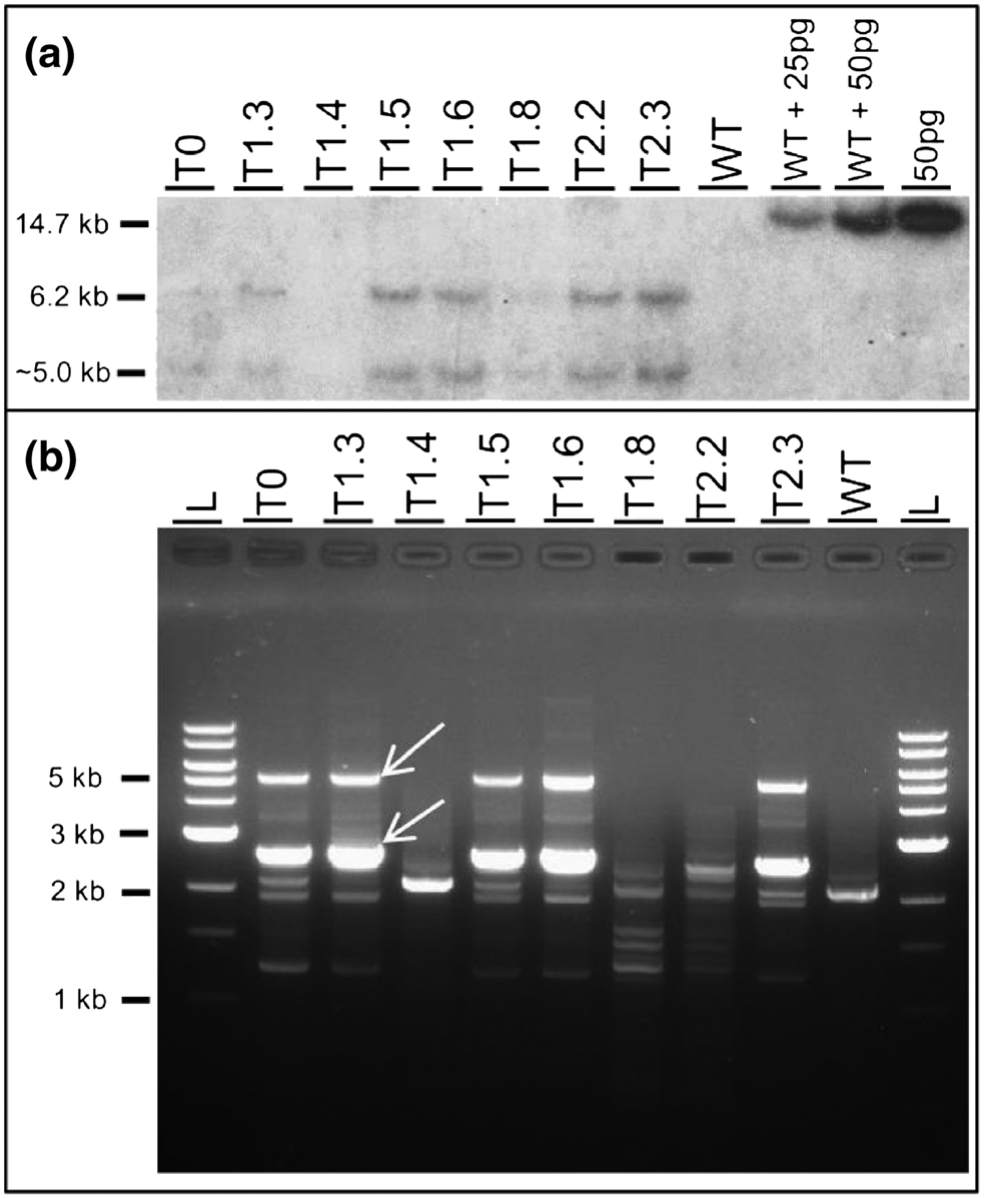
(a) Southern blot (b) TAIL‐PCR analyses for T_0_ plant VPZ‐23, five segregating T_1_ plants, two homozygous T_2_ plants and wild type control (WT). The final three lanes show 25 and 50 pg digested VPZ plasmid DNA with 10 *μ*g of digested WT DNA and 50 pg of VPZ plasmid without WT DNA. Arrows in panel b indicate the bands that were absent in WT and show a size shift between reaction 2 and 3 in the TAIL‐PCR and scored in Table 1. TAIL‐PCR was performed with AD3 and T‐DNA specific primers RB3.

Southern blot analysis on selected VPZ‐23 plants showed two bands of approximately 5.0 and 6.2 kb in the VPZ‐23 T_0_ plant (lane 1), suggesting two T‐DNA copies present in the primary transformant (Fig. 1a; Table 1). Self‐pollination resulted in T_1_ progeny with zero (T_1.4_), two T‐DNA copies (T_1.3_ and T_1.8_) or four copies (T_1.5_ and T_1.6_). TAIL‐PCR was performed on the same plants using AD1, AD2 and AD3 primers with increasing degeneracy (64‐, 128‐ and 256‐fold, respectively) (Table 1, Fig. 1b and Fig. S2). Results with AD1 showed only one unique band for plants T_0_, T_1.3_, T_1.5_, T_1.6_ and T_2.3_, whereas the second band expected based on Southern blot results was missing and DNA from plants T_1.8_ and T_2.2_ failed to generate any unique band. Increasing the degeneracy of the primer to 256‐fold resulted in one unique band for plants T_1.8_ and T_2.2_, and two unique bands for plants T_0_, T_1.3_, T_1.5_, T_1.6_ and T_2.3_. The corresponding T‐DNA copy number from qPCR showed no copies in WT and T_1.4_, and clear differences between plants T_0_, T_1.3_ and T_1.8_ (2.17–2.37), and plants T_1.5_, T_1.6_, T_2.2_ and T_2.3_ (4.4–5.37) (Table 1). However, in general the T‐DNA copy numbers were higher than Southern blot estimates by 0.17 to 1.37, with standard errors ranging from 0.06 (T_0_) to 0.62 (plant T_1.8_). Finally, T‐DNA copy number estimates from ddPCR were very closely approximated to the corresponding Southern blot results with 1.83–1.90 for plants with two copies and 3.74–4.24 for plants with four copies. In contrast to qPCR results, no systematic over‐estimation was present in the ddPCR results relative to the Southern blot analysis. Standard errors were generally lower for ddPCR than qPCR, ranging between 0.04 (plants T_1.5_ and T_1.8_) to 0.28 (plant T_1.6_) even though only two technical replicates were used compared to four for qPCR, thus more strongly reflecting the likelihood of two copies per locus as visualized by the Southern hybridization (Fig. 1; Table 1).

**Table 1:** Comparison of the T‐DNA copy numbers estimated by Southern blot, qPCR, TAIL‐PCR and ddPCR for eight plants representing three generation (T_0_, T_1_ and T_2_) and corresponding control wild type (WT) of *N. tabaccum*. For TAIL‐PCR the T‐DNA copy number was assessed with the use of three different reaction sets with primers differing in degeneration: AD1, AD2 and AD3, with 64‐, 128‐ and 256‐fold degeneracy, respectively. For qPCR and ddPCR the T‐DNA copy number was derived from the estimated sample concentration of T‐DNA relative to actin (± se; *n* = 4 for qPCR; *n* = 2 for ddPCR).

	T‐DNA copy number					
	Southern blot	qPCR	TAIL‐PCR			ddPCR
Plant ID			AD1	AD2	AD3	
VPZ‐23 T_0_	2	2.37 ± 0.06	1	1	2	1.83 ± 0.11
VPZ‐23 T_1.3_	2	2.17 ± 0.18	1	1	2	1.90 ± 0.20
VPZ‐23 T_1.4_	0	0 ± 0.00	0	0	0	0 ± 0.00
VPZ‐23 T_1.5_	4	4.83 ± 0.19	1	1	2	3.74 ± 0.04
VPZ‐23 T_1.6_	4	5.37 ± 0.15	1	1	2	4.21 ± 0.28
VPZ‐23 T_1.8_	2	2.29 ± 0.62	0	1	1	1.87 ± 0.04
VPZ‐23 T_2.2_	4	4.40 ± 0.12	0	0	1	4.24 ± 0.11
VPZ‐23 T_2.3_	4	4.72 ± 0.16	1	1	2	4.18 ± 0.16
WT	0	0 ± 0.00	0	0	0	0 ± 0.00

### Reproducibility of ddPCR and qPCR estimates

Because qPCR and ddPCR appeared to have greater accuracy than TAIL‐PCR, an additional side‐by‐side comparison of these two methods was performed to evaluate reproducibility. Using DNA from a plant transformed with the VPZ construct, two different primer sets targeted to contrasting regions per gene (*At*VDE, *At*PsbS and *At*ZEP) were used, together with primers targeting regions in two different reference sequences (α‐tubulin and actin). These reference sequences have different copy numbers in the tobacco genome, two versus four, respectively. The qPCR efficiency for these eight primer sets ranged from 97 to 102%. The resulting 12 combinations of transgene and reference primer sets and the corresponding estimated copy number using qPCR and ddPCR are shown in Table 2. T‐DNA copy number estimates varied between 1.49 and 5.38 for qPCR but only 1.82 and 2.26 for ddPCR. Estimates using α‐tubulin as a reference sequence appeared consistently higher when determined by qPCR, whereas no obvious bias could be detected for either actin or α‐tubulin in ddPCR. As in the previous comparison, standard errors for the estimated T‐DNA copy numbers were generally higher for qPCR (0.05–0.49) compared to ddPCR (0.02–0.12), even though fewer technical replicates were used for ddPCR; two versus four. Because all analyses should arrive at the same answer, we computed the variance across the 12 combinations per method in order to evaluate reproducibility. The variance across the qPCR determinations was substantially higher than across the ddPCR estimates, 1.10 versus 0.02.

**Table 2:** Comparison of T‐DNA copy number estimated using ddPCR or qPCR estimations of PCR template concentration of six different primersets targeting T‐DNA regions and either actin or α‐tubulin as a reference gene. Analysis was performed on digested DNA of VPZ‐46 T_1.5_ plant carrying a T‐DNA with three genes (*At*VDE, *At*Psbs and *At*ZEP). (± se; *n* = 4 for qPCR; *n* = 2 for ddPCR).

	qPCR		ddPCR	
	T‐DNA copy number		T‐DNA copy number	
Primer set name	actin	α‐tubulin	actin	α‐tubulin
AtPsbS_3	1.49 ± 0.11	2.85 ± 0.25	2.01 ± 0.05	1.82 ± 0.03
AtPsbS_4	1.86 ± 0.49	2.29 ± 0.06	2.19 ± 0.06	1.98 ± 0.03
AtVDE_1	2.08 ± 0.26	3.19 ± 0.13	2.26 ± 0.12	2.05 ± 0.06
AtVDE_4	3.51 ± 0.20	5.38 ± 0.10	2.06 ± 0.05	1.87 ± 0.02
AtZEP_1	2.01 ± 0.18	3.08 ± 0.09	2.16 ± 0.08	1.96 ± 0.04
AtZEP_4	1.92 ± 0.09	2.94 ± 0.05	2.16 ± 0.11	1.96 ± 0.06
Variance per method	1.10		0.02	

### What is the most suitable method to assess T‐DNA copy number and homozygosity?

Table 3 shows that all four methods can generate T‐DNA copy number estimates in T_0_ transformants, although the precision is low for qPCR and TAIL‐PCR. Additionally, Southern blot analysis, qPCR and ddPCR were effective in estimating loci number and identifying homozygous offspring in analysis of T_1_ progeny. Southern blots were taken as the benchmark for precision in determining copy number. Although both qPCR and ddPCR matched Southern blot determinations, qPCR gave a greater variance and also tended to over‐estimate copy number. TAIL‐PCR clearly under‐estimated copy number, and this was improved little by use of the most degenerate primer, AD3. The comparison between ddPCR and qPCR using a set of 12 different combinations of T‐DNA and reference sequence primer sets, showed good reproducibility in the ddPCR estimates, whereas qPCR results were much more variable, leading to contrasting results between the different primer set combinations (Table 2). A key consideration for all methods is the time that they require. TAIL‐PCR requires three consecutive PCR programmes, which all have to be verified using gel electrophoresis, and together add up to approximately 12 h. Both qPCR and ddPCR protocols made use of pre‐digested DNA, which was done overnight; hence, the total protocol as described here would take up to one day. The estimate for Southern blot analysis is considerably longer, accounting for the exposure time of the x‐ray film to the labelled membrane. In addition, all PCR methods can be easily automated using 96‐well plates or greater, allowing many samples to be run in this time. The largest Southern blot gel would typically be 18 sample tracks; this could be multiplied but automation is considerably more difficult (Table 3).

**Table 3:** Comparison of time required, precision, reproducibility and form of the output data of four different methods used in T‐DNA copy estimation.

		Method			
Feature		Southern blot	qPCR	TAIL‐PCR	ddPCR
Time required^b^		4–7 days	1.5 day^a^	2 days	1.5 day^a^
Precision		High	Low	Low	High
Reproducibility		n.e.^c^	Low	n.e.^c^	High
Type of output data	T‐DNA copy number	+	+	−/+	+
	T‐DNA locus number	+	+	−	+
	Homozygosity of T_1_ plants	−/+	+	−	+

a It is possible to make these protocols faster by shortening the pre‐digestion time.

b Estimate does not include the additional time to generate T_1_ progeny, which will be very species dependent.

c Not estimated.

### Screening for homozygous plants in T_1_ progeny

Because ddPCR results were highly reproducible and consistent with Southern blot analysis, we further evaluated ddPCR as a means to rapidly identify homozygosity in T_1_ progeny. Because T_0_ plants are hemizygous, cross‐comparison between T_0_ and T_1_ T‐DNA copy numbers allows easy identification of homozygous plants in T_1_, from which the WT segregants had already been eliminated by glufosinate ammonium treatment. In *N. tabacum*, which is tetraploid but a functional diploid, homozygous plants in the T_1_ offspring should show a doubling of the T‐DNA copy number relative to the corresponding T_0_ plant. Using this criterion, Table 4 shows that ddPCR provides an unambiguous identification of the homozygotes in the 32 T_1_ progeny.

**Table 4:** Results of screening for copy number to identify homozygous plants in T_1_ segregating progenies with the use of ddPCR. Seedlings of T_1_ lines were sprayed with glufosinate ammonium solution before single plants were transferred to pots and grown for tissue collection for ddPCR analyses. Bold numbers indicate homozygous plants identified by a duplication of the copy number in the corresponding T_0_ plant (± se; *n* = 2).

Generation	Transformation event			
	PsbS‐43	PsbS‐46	VPZ‐29	VPZ‐34
T_0_	1.09 ± 0.13	0.98 ± 0.18	0.97 ± 0.09	0.97 ± 0.14
T_1_	**2.10 ± 0.22**	**2.04 ± 0.16**	**1.96 ± 0.16**	0.94 ± 0.21
	**2.13 ± 0.20**	1.08 ± 0.11	0.93 ± 0.15	**1.93 ± 0.06**
	**2.05 ± 0.04**	1.01 ± 0.28	0.98 ± 0.16	**2.08 ± 0.06**
	1.12 ± 0.25	**2.08 ± 0.10**	1.04 ± 0.20	0.98 ± 0.09
	**2.02 ± 0.16**	**2.09 ± 0.08**	1.03 ± 0.27	**1.80 ± 0.21**
	**2.04 ± 0.05**	**2.04 ± 0.05**	0.95 ± 0.08	**1.87 ± 0.11**
	**2.26 ± 0.04**	**2.10 ± 0.08**	1.03 ± 0.10	**1.80 ± 0.31**
	**2.10 ± 0.28**	1.08 ± 0.26	1.02 ± 0.03	**2.02 ± 0.20**

### Comparison of T‐DNA copy numbers and numbers of T‐DNA loci

**Figure 2:**
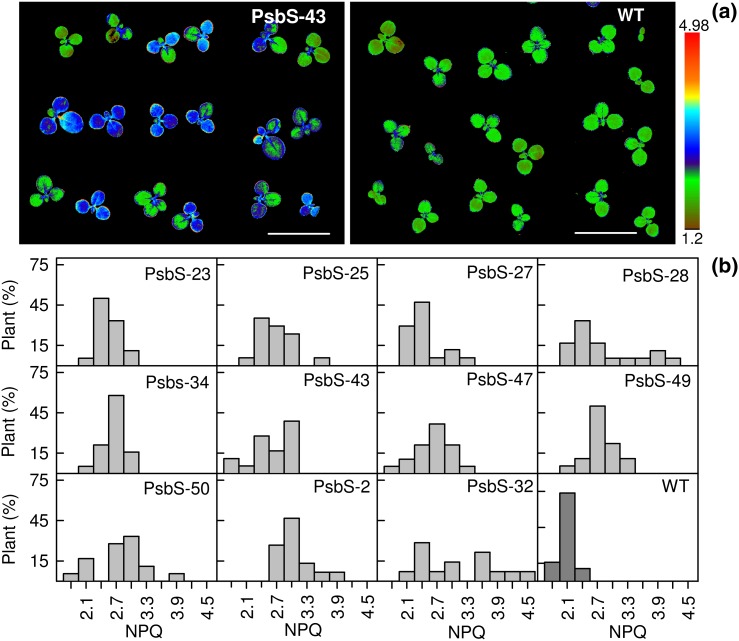
The segregation of nonphotochemical quenching (NPQ) in 10‐day‐old seedlings transformed by *Nb*Psbs plants of *N. tabacum*. (a) Imaged NPQ for PsbS‐43 T_1_ (segregating T_1_ progeny of T_0_ plant carrying one T‐DNA copy) and wild type control (WT); (b) distribution of NPQ in 11 T_1_ segregating populations and WT. Presented values of NPQ were recorded after 10 min of induction at 1000 *μ*mol quanta m^‐2^ s^‐1^. Bar on panel (a) represents 2.5 cm.

Assuming Mendelian inheritance, segregation analysis is another means to obtain homozygous lines; however, if multiple insertions occur at the same locus or are otherwise linked, this will fail to show true copy number. For 26 independent transformations, 17 of these showed approximately 1:3 segregation of glufosinate ammonium resistance, indicating a single T‐DNA insertion locus. However, ddPCR showed that five of these 17 carried more than one T‐DNA copy, and in one case 5. This suggests a high frequency of multiple inserts at a single locus that would not be detected by segregation analysis (Table 5). Chlorophyll fluorescence imaging of NPQ was used as an easily screenable phenotype in assessing the PsbS transformant segregation, of which an example is shown in Fig. 2a. PsbS transformants were easily distinguishable from WT based on increased NPQ (Fig. 2b), and segregation analysis gave very similar locus number results compared to glufosinate ammonium resistance (Table 5).

**Table 5:** T‐DNA copy (± se; *n* = 2) estimated by digital droplet PCR (ddPCR) and estimated number of T‐DNA loci based on segregation analyses (Chi^2^‐test for 1:3 segregation, *α* = 0.1). Segregation was scored based on either resistance to glufosinate ammonium (GA) in 17‐day‐old seedlings or level of non‐photochemical quenching (NPQ) in 7‐day‐old seedlings. NPQ values were induced by 10 min exposure to PFD of 1000 *μ*mol quanta m^‐2^ s^‐1^.

	ddPCR	Segregation of resistance to GA leaf paint in T_1_ generation			Segregation based on NPQ in Fig. 2b		
ID of T_0_ plant	No. of T‐DNA copy	No. of susceptible plants (%)	No. of resistant plants (%)	No. of T‐DNA loci in line	No. of plants with WT NPQ (%)	No. of plants with NPQ higher than WT (%)	No. of T‐DNA loci in line
PsbS‐23	0.99 ± 0.17	6 (27)	16 (73)	1	2 (11)	16 (89)	1
PsbS‐25	0.98 ± 0.06	8 (33)	16 (67)	1	2 (12)	15 (88)	1
PsbS‐27	0.97 ± 0.04	5 (23)	17 (77)	1	7 (41)	10 (59)	1
PsbS‐28	1.04 ± 0.10	5 (21)	19 (79)	1	3 (17)	15 (83)	1
PsbS‐34	0.87 ± 0.22	4 (17)	20 (83)	1	1 (5)	18 (95)	>1
PsbS‐43	1.09 ± 0.13	5 (21)	19 (79)	1	4 (22)	14 (78)	1
PsbS‐47	0.98 ± 0.18	3 (12.5)	21 (87.5)	1	4 (21)	15 (79)	1
PsbS‐49	1.99 ± 0.21	0 (0)	23 (100)	>1	1 (6)	17 (94)	>1
PsbS‐50	1.97 ± 0.10	3 (12.5)	21 (87.5)	1	4 (22)	14 (78)	1
PsbS‐2	3.94 ± 0.21	0 (0)	24 (100)	>1	0 (0)	15 (100)	>1
		1 (1), a	71 (99)^a^	>1^a^			
PsbS‐32	3.89 ± 0.05	6 (26)	17 (74)	1	4 (29)	10 (71)	1
VPZ‐14	1.03 ± 0.38	5 (21)	19 (79)	1	na	na	na
		19 (26)^a^	53 (74)^a^	1^a^			
VPZ‐29	0.97 ± 0.09	6 (26)	17 (74)	1	na	na	na
VPZ‐31	1.07 ± 0.09	5 (21)	19 (79)	1	na	na	na
VPZ‐34	0.97 ± 0.14	8 (33)	16 (67)	1	na	na	na
VPZ‐38	1.02 ± 0.05	2 (9)	21 (91)	>1	na	na	na
VPZ‐54	0.97 ± 0.07	9 (37.5)	15 (62.5)	1	na	na	na
VPZ‐13	2.12 ± 0.08	5 (23)	17 (77)	1	na	na	na
		20 (28)^a^	51 (72)^a^	1^a^			
VPZ‐23	1.83 ± 0.11	4 (17)	20 (83)	1	na	na	na
		18 (25)^a^	54 (75)^a^	1^a^			
VPZ‐28	1.91 ± 0.16	2 (8)	22 (92)	>1	na	na	na
VPZ‐35	2.94 ± 0.13	1 (4)	23 (96)	>1	na	na	na
VPZ‐50	2.86 ± 0.05	1 (4)	23 (96)	>1	na	na	na
VPZ‐52	2.88 ± 0.17	2 (8)	22 (92)	>1	na	na	na
VPZ‐51	4.83 ± 0.14	4 (17)	20 (83)	1	na	na	na
VPZ‐33	11.96 ± 0.13	15 (62.5)	9 (37.5)	na^b^	na	na	na
VPZ‐36	12.09 ± 0.08	0 (0)	24 (100)	>1	na	na	na

a Data collected of resistance to leaf‐paint of GA solution in 72 field‐grown plants.

b Not estimated because of non‐Mendelian segregation. na – not applicable.

## Discussion

### Evaluation of different methods to establish T‐DNA copy number

Although Southern blots have historically been used to estimate transgenic allele loci and copy number, the method typically takes at least a week to generate results, relatively few samples can be processed in parallel and require specialized radiation laboratories. The PCR‐based methods presented in the current manuscript took typically less than two days, can be easily optimized to quantify final copy number in a matter of hours and can easily be automated. Therefore, PCR‐based methods are attractive because of reduced time to results and general ease of use. Using Southern blots as a benchmark, we show that T‐DNA loci determination and estimate of copy numbers predicted with the ddPCR method (Table 1) were most accurate across a subset of three generations of transgenic plants, with contrasting zygosity and copy number and generally showed the lowest variance in estimates. Estimates from qPCR were more variable, and consistently overestimated copy numbers relative to the Southern blot estimates, while TAIL‐PCR underestimated copy number (Table 1). Similar problems have been shown for copy number estimation based on inverse PCR, which is also based on amplification of flanking sites (Does *et al.* 1991). In TAIL‐PCR, closely spaced T‐DNA may decrease the statistical probability of a suitable binding site for the degenerate primer between subsequent T‐DNA insertions. If this was the case, using a higher fold of degeneracy should improve the number of amplified flanking sites. In six of the assessed samples, using a more degenerate primer indeed did improve the number of bands identified (see AD1 to AD3, Table 1), but in two samples the number of bands was still lower than expected based on Southern blot results. Therefore, TAIL‐PCR may be used reliably to detect at least one T‐DNA copy, but the frequent underestimation relative to ddPCR and Southern blot estimates is difficult to troubleshoot when *a priori* knowledge of the correct copy number is not already available.

In a separate comparison, ddPCR was also shown to be more reproducible than qPCR in a parallel analysis of 12 different combinations of target and reference primer sets on DNA from a single plant (Table 2). Results for ddPCR consistently indicated two T‐DNA copies, independent of the primer combination used. However, qPCR estimates showed a range of contrasting T‐DNA copy numbers, varying between 1.49 and 5.38, such that interpretation of the T‐DNA copy number was unreliable and tended to over‐estimation. These results can be explained by a number of advantages favouring ddPCR over qPCR for the purpose of T‐DNA copy number estimation. Contrary to qPCR, in ddPCR the level of fluorescence is not directly coupled to the PCR target quantification, allowing PCR efficiency requirements to be less strict. As a result ddPCR is more robust against factors interfering with PCR target amplification and no standard curve is required. The linearity of the ddPCR technique also allows precise detection of small fold changes in copy concentration, whereas the logarithmic detection scale of qPCR limits the capabilities to accurately detect small differences. A number of medical studies have recently compared qPCR and ddPCR for absolute quantification of human microRNAs and copy number variation. These studies also found ddPCR to have far less variability and increased accuracy compared to qPCR (Hindson *et al.* 2013; Bharuthram *et al.* 2014).

### T‐DNA copy number and number of independent inherited loci

Because T_0_ plants carrying a single T‐DNA locus are most easily progressed to a fully homozygous generation, in most cases these primary transformants are preferred for further study. Table 4 shows that homozygous individuals in four lines with a single T‐DNA can be easily distinguished. The ratio of homozygous versus hemizygous T_1_ progeny in Table 4 is slightly higher than the expected 1:2. Because the T_1_ offspring was first treated with glufosinate ammonium solution to eliminate the WT fraction, this offset may be explained by a possible *bar* gene dosage effect between the hemizygous and homozygous lines, possibly favouring the latter to be identified for further screening.

The consistency of predictions between independent data for the well‐established selectable marker glufosinate ammonium resistance and NPQ levels in the PsbS transformants (Table 5) shows that co‐transformation of the PsbS gene can be used as a visual marker, which avoids issues with toxicity and resistance outcrossing often associated with traditional markers (Miki & McHugh, 2004). In 5 out of 26 T_0_ plants, copy numbers assessed by ddPCR were higher than the loci number estimated by segregation analyses of glufosinate ammonium resistance and NPQ. These T_0_ plants were all estimated to have a single independent T‐DNA locus based on segregation analyses (Table 5), whereas ddPCR results showed up to five T‐DNA copies (VPZ‐51) were integrated. These findings may be explained by the presence of silent or incomplete T‐DNA copies, which would be detected by ddPCR but not affect the inheritance pattern of the selectable marker. Alternatively, these results suggest a relatively high occurrence of multiple T‐DNA copies per locus (at least 19% in our study), which is consistent with earlier reports (Jorgensen *et al.* 1987; Kohli *et al.* 1999; Gelvin 2003) and shows the importance of routinely checking the T‐DNA copy number, even if segregation analysis suggests the occurrence of only one T‐DNA containing locus. Because multiple T‐DNA copies at the same locus can increase the occurrence of silencing, these findings exemplify the importance to allow precise and routine selection of single copy T_0_ transformants for further analysis, which cannot be substituted by segregation analysis. We used two different constructs, with contrasting T‐DNA lengths (4.5 kb and 10.8 kb for PsbS and VPZ, respectively), but the length of the T‐DNA seemed to make little difference in the occurrence of multiple T‐DNA insertions.

The iterative cycle of modification, testing and evaluation which is essential in genetic engineering approaches relies on high‐throughput generation and verification of transgenic plants. Our results show ddPCR to be accurate, precise and fast in determining T‐DNA copy numbers for screening the high numbers of transgenic plants being produced today across a wide range of research goals. The protocol (Supporting Information; Appendix 1) can also easily be modified to allow automated high‐throughput screening of DNA samples. Our study shows serious limitations in applying qPCR and TAIL‐PCR in estimating copy number. The results also show a high frequency of genetically linked insertions which could not be detected by segregation analyses, emphasizing the importance of routinely checking T‐DNA copy numbers in generation of transgenic plants. ddPCR provides a high‐throughput means to achieve this.

## Supporting information

Additional Supporting Information may be found in the online version of this article at the publisher's web‐site
**Table S1.** Sequence of primers used in ddPCR, qPCR and TAIL‐PCR to assess copy number of T‐DNA inserts.
**Figure S1.** Maps of T‐DNA of PsbS and VPZ constructs.
**Figure S2.** Agarose gel of primary, secondary and tertiary TAIL‐PCR products.
**Appendix 1.** ddPCR protocol.docx

Supporting info item

Supporting info item

Supporting info item

Supporting info item
