# Supplementary material for: An evaluation of new and established methods to determine T‐DNA copy number and homozygosity in transgenic plants
Source: Plant Cell Environ. 2016 Jan 21;39(4):908–17. doi: 10.1111/pce.12693 (PMC5021166; doi:10.1111/pce.12693)
Supplement: Supplementary file 1 — Supporting info item [file PCE-39-908-s001.docx]

**Table S1** Sequence of primers used in ddPCR, qPCR and TAIL-PCR to assess copy number of T-DNA inserts in genome of *N. tabacum* and verified the transgene expression

| Sequence name in which primers bind | Primer name | Primer sequence | | Amplicon length (bp) |
| --- | --- | --- | --- | --- |
|  |  | Forward (5’-3’) | Reverse (5’-3’) |  |
| **ddPCR and qPCR** |  |  |  |  |
| *A. thaliana* PsbS | AtPsbS_3 | TCGTTGGTCGTGTTGCTATG | CTCTGCTTCGTAAATCGGTATCC | 119 |
|  | AtPsbS_4 | GCACTACCATCTAGGAGACAATC | CCATCCTCAACCTTGCTCTT | 107 |
| *A. thaliana* VDE | AtVDE_1 | GGAGATCCTCACGTCCTTTATC | TCAGCTCTTTCAGCTCATTCT | 99 |
|  | AtVDE_4 | AGACAGTGGAAGAAGGTGAAAG | CTGGAACAAGGTCATCTCAGTC | 110 |
| *A. thaliana* ZEP | AtZEP_1 | TACAGAGGCCCGATTCAAATAC | TGATACACCCAGCTTCCATAAC | 97 |
|  | AtZEP_4 | CTCACTGACAAAGCCGATGA | GTCGCCGTGTGGAATTAGATA | 102 |
| *N. benthamiana* PsbS and *A. tumefaciens* HSP1 terminator | NbPsbS-linker_1 | CTGATGAGGAAGAGGAGTGAAC | AAAGCAGGACTCTAGGGACTA | 104 |
| *N. tabacum* actin | NtActin_1 | CCTCACAGAAGCTCCTCTTAATC | ACAGCCTGAATGGCGATATAC | 105 |
| *N.* *tabacum*  α tubulin | NtTubulin­­_1 | GTACATGGCCTGTTGTTTGATG | CTGGATGGTCCTCTTTGTCTTT | 94 |
| **TAIL-PCR** |  |  |  |  |
| na | AD1 | NTCGA(G/C)T(A/T)T(G/C)G(A/t)GTT |  | na |
| na | AD2 | NGTCGA(G/C)(A/T)GANA(A/T)GAA |  | na |
| na | AD3 | (A/T)GTGNAG(A/T)ANCANAGA |  | na |
| 373bp from RB of T-DNA | RB1 | AAGTTACGGGCACCATTCA |  | na |
| 256bp from RB of T-DNA | RB2 | ACCTTGACAGTGACGACAAATC |  | na |
| 123bp from RB of T-DNA | RB3 | CCTGGCGTTACCCAACTTAATC |  | na |
|  | | | | |

VDE -violaxanthin de-epoxidase

ZEP - zeaxanthin epoxidase

PsbS - photosystem II subunit S

HSP1 - heat shock protein 1

RB - right border

na - not applicable
