# Supplementary figures and images for: An evaluation of new and established methods to determine T‐DNA copy number and homozygosity in transgenic plants
Source: Plant Cell Environ. 2016 Jan 21;39(4):908–17. doi: 10.1111/pce.12693 (PMC5021166; doi:10.1111/pce.12693)

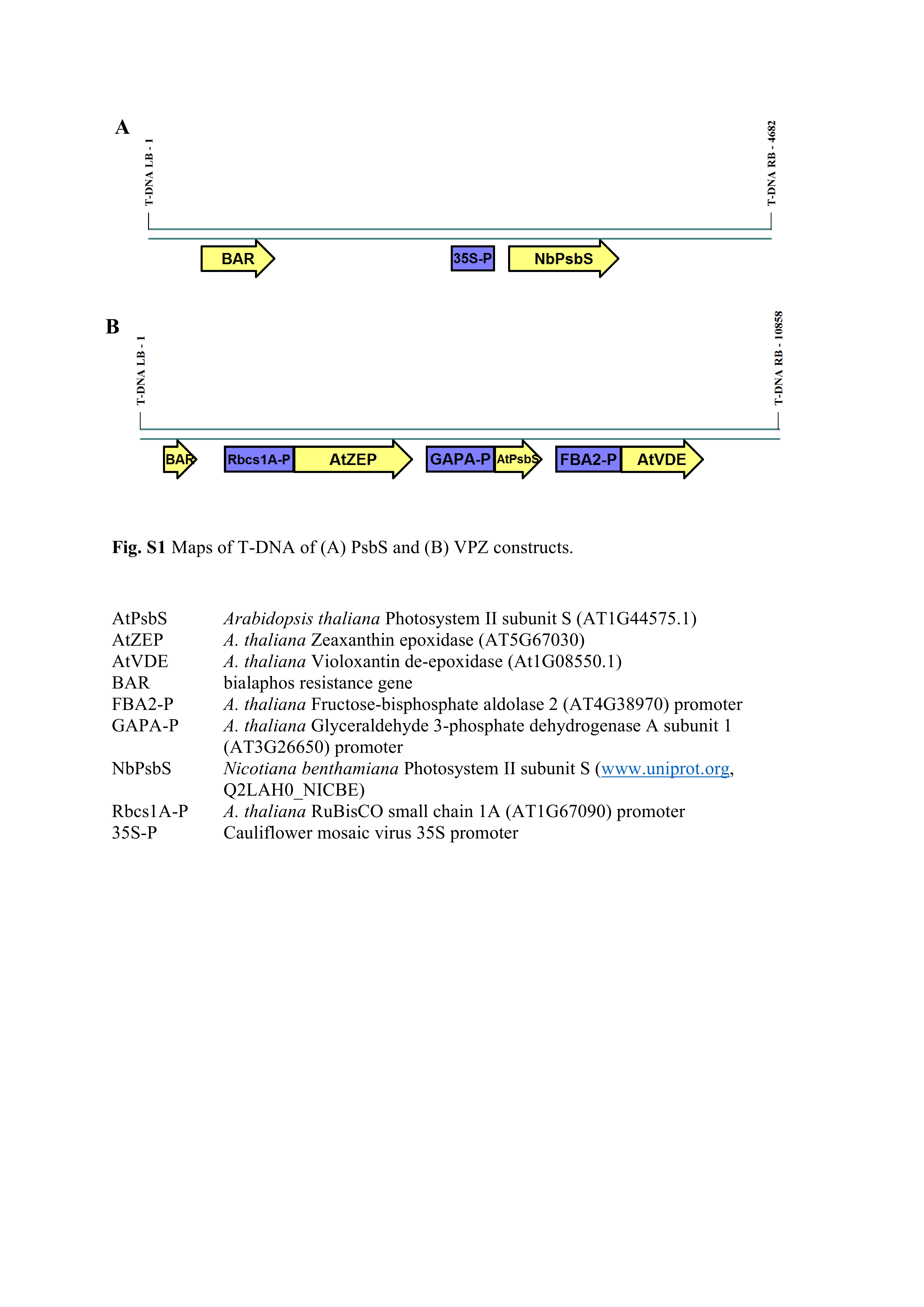

Supplement: Supplementary file 2 — Supporting info item [file PCE-39-908-s002.jpg]

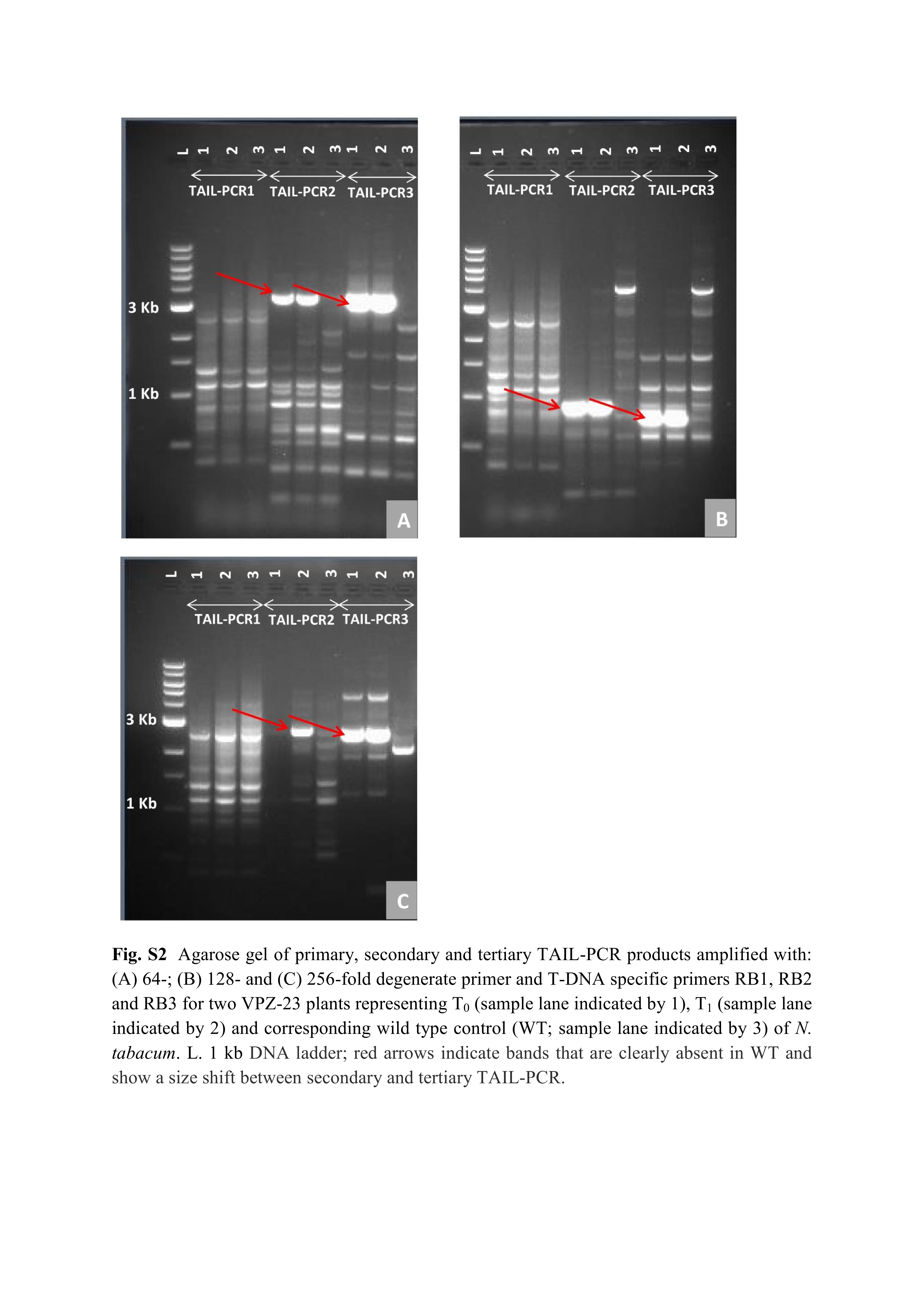

Supplement: Supplementary file 3 — Supporting info item [file PCE-39-908-s003.jpg]
