# Supplementary material for: An evaluation of new and established methods to determine T‐DNA copy number and homozygosity in transgenic plants
Source: Plant Cell Environ. 2016 Jan 21;39(4):908–17. doi: 10.1111/pce.12693 (PMC5021166; doi:10.1111/pce.12693)
Supplement: Supplementary file 4 — Supporting info item [file PCE-39-908-s004.docx]

**Supplemental Material Appendix 1**

**ddPCR protocol.docx**

**Preparing DNA by digestion**

1. Prepare 50 µL DNA dilutions (200ng /µL) for each sample. Use MilliQ autoclaved water (dMQ H_2_O) to elute DNA.
2. Transfer 10 µL of diluted DNA to microtubes/ PCR plate.
3. Set up the **digestion of DNA**

1x

Digestion enzyme 1µL (20U)

Cut smart buffer 5µL

dMQH2O (50µL -x)

2000ng of DNA x

1. Incubate the digestion mix for 17 h at 37 ^o^C.
2. Transfer digested DNA to separate 1.5ml tubes.
3. Purify the DNA with cleaning and concentrating columns (D4014; Zymo Research, Irvine, CA, USA) according to the manufacturer instructions.
4. Elute the clean DNA with 55 µL of dMQ H_2_O.

**General comments:**

- Make sure that the digestion enzyme does not cut inside the PCR amplicon.
- To minimize the occurrence of secondary structures and increase the accessibility of the template the fragment of DNA carrying the PCR-amplicon should be shorter than 2200bp.
- Overnight digestion is recommended for relatively large genomes to reduce the fluorescence amplitude of negative droplets (background), in which case it is recommended to use high fidelity digestion enzymes to avoid off-target cuts.

**Preparing the ddPCR mix**

1. For each of the samples, transfer 11.5 µL of digested and cleaned DNA to a 96-well PCR plate or micro-tube. Use at least 2 technical replicates for both T-DNA and reference sequence ddPCR reactions.
2. Prepare the master mix

EvaGreen supermix 2x 12.5 µL

Primer Forward 0.25 µL (100 nM)

Primer Reverse 0.25 µL (100 nM)

dMQH_2_O 0.50 µL

13.5 µL

1. Add 13.5 μL of master mix to each sample.

**Generating droplets:**

1. Insert a cartridge (186-4007, Bio-Rad, Hercules, CA, USA) in cartridge holder (186-3051, Bio-Rad, Hercules, CA, USA). Gently mix by pipetting, before transferring 20µL of each mastermix + sample to the sample row. Use 2-20 µL tips with multichannel pipette and be careful not to create bubbles.
2. Add 70 µL of QX200 droplet generation oil for EvaGreen (186-4005, Bio-Rad, Hercules, CA, USA) to the oil row on the cartridge. Use 200 µL tips with multichannel pipette.
3. Put the gasket on top of the cartridge.
4. Insert holder + cartridge + gasket in droplet generator.
5. After the droplets are generated, transfer 40µL of droplets to a new plate (PCR semi-skirted plate; 951020362, Eppendorf, Enfield, CT, USA). Use 30-300µL pipette tips and create a 45° angel between bottom of the well and tips end. Pipetting should be done very slowly to avoid damaging the droplets.
6. Cover the plate by foil seal (Pierceable foil heat seal, 181-4040; Bio-Rad, Hercules, CA, USA) and seal for 5s at 180 ºC in plate sealer (Px1 PCR plate sealer, 181-4000; Bio-Rad, Hercules, CA, USA).

**PCR reaction**

1. Run the reaction in a deep-well thermal cycler (e.g. C1000 Touch 185-1196, Bio-Rad, Hercules, CA, USA) at the following PCR program:

Enzyme activation 95ºC 5 min

Denaturation 95ºC 30s

Annealing/extension 56.5ºC 1.30 min 40x ramp

rate 2 ºC/sec

Signal stabilization 4ºC 5 min

90ºC 5 min

Hold 12 ºC infinite

**General comments:**

- Adjust the annealing/extension temperature to primer melting temperature

**Droplet reading**

1. Immediately after the PCR reaction is finished, load the plate in the droplet reader (QX200, Bio-Rad, Hercules, CA, USA) to analyze the individual fluorescence levels per droplet.

**Specific application of the ddPCR protocol to sequences used in the present manuscript**

**PsbS construct**

High similarity of *N. tabacum* PsbS to *N. benthamiana* PsbS made it impossible to design primers specific to one of the PsbS genes. Therefore a primer set was designed with the forward primer annealing to *Nb*PsbS and the reverse primer to a sequence in the terminator *A. tumefaciens* heat shock protein 1 (primer set: NbPsbS-linker_1; see Table S1). As a negative control, the NbPsbS-linker_1 primer set was tested on wild type (WT) plants of *N. tabacum* and showed no amplification. DNA for PsbS transformants was digested simultaneously by *Sac*I (R3156, New England Biolabs, Ipswich, MA, USA) and *Hind*III (R3104, New England Biolabs, Ipswich, MA, USA) enzymes giving a DNA fragment of 1338 bp containing the amplicon amplified by the NbPsbS-linker_1 primers.

**VPZ construct**

For the VPZ construct, 6 primer sets were designed (2 for each of the 3 genes) and tested on the same DNA as given in Table 2 (see Table S1 for sequences). All primer sets yielded similar results, and the AtVDE_4 primer set was used for routine copy number estimation throughout, unless otherwise stated (Table S1). DNA samples for VPZ transformants were digested by *Hind*III enzyme which resulted in several fragments shorter than 2200bp containing the amplicons for each primer set.
